# Oenothein B, a Bioactive Ellagitannin, Activates the Extracellular Signal-Regulated Kinase 2 Signaling Pathway in the Mouse Brain

**DOI:** 10.3390/plants10051030

**Published:** 2021-05-20

**Authors:** Satoshi Okuyama, Yoshiko Furukawa, Morio Yoshimura, Yoshiaki Amakura, Mitsunari Nakajima, Takashi Yoshida

**Affiliations:** 1 Department of Pharmaceutical Pharmacology, College of Pharmaceutical Sciences, Matsuyama University, 4-2 Bunkyo-cho, Matsuyama 790-8578, Ehime, Japan; sokuyama@g.matsuyama-u.ac.jp (S.O.); mnakajim@g.matsuyama-u.ac.jp (M.N.); 2 Department of Pharmacognosy, College of Pharmaceutical Sciences, Matsuyama University, 4-2 Bunkyo-cho, Matsuyama 790-8578, Ehime, Japan; myoshimu@g.matsuyama-u.ac.jp (M.Y.); amakura@g.matsuyama-u.ac.jp (Y.A.); xp769b@bma.biglobe.ne.jp (T.Y.); 3 Department of Pharmaceutical Sciences, Okayama University, 1-1-1 Tsushima-naka, Kita-ku, Okayama 700-8530, Japan

**Keywords:** oenothein B, ellagitannin, neuroprotection, extracellular signal-regulated kinase (ERK), cAMP response element-binding protein (CREB), brain

## Abstract

(1) Background: Oenothein B, a cyclic dimeric ellagitannin present in various medicinal plants, has been reported to exert diverse effects that are beneficial for the treatment and prevention of diseases, including cancer and infections. We recently showed that oenothein B also functions in the brain because its oral administration to systemic inflammatory model mice reduced inflammatory responses in the brain and suppressed abnormal behavior. (2) Results: The present in vivo results demonstrated that oenothein B activated extracellular signal-regulated kinase 2 and cAMP response element-binding protein in the brain, both of which play important roles in synaptic transmission and learning/memory in the central nervous system (CNS). (3) Conclusions: These results suggest that oenothein B exerts neuroprotective effects on the CNS by not only its anti-inflammatory activity but also by enhancing neuronal signaling pathways.

## 1. Introduction

**Figure 1:**
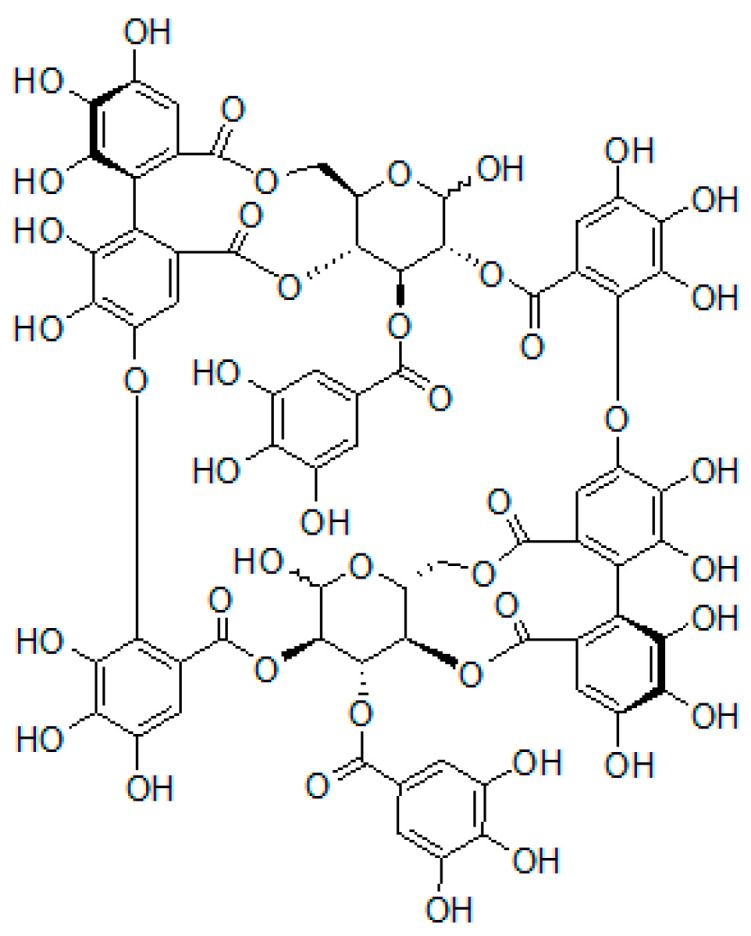
Structure of oenothein B.

Oenothein B, which has a unique macrocyclic structure (Figure 1), was initially isolated from the leaves of *Oenothera erythrosepala* (Oenotheraceae) in 1990 [1] and was subsequently found to be widely distributed in various medicinal plants of Myrtaceae and Lythraceae other than Oenotheraceae [2]. In vitro and in vivo studies demonstrated that oenothein B exerted various biological effects, including antioxidant, anti-inflammatory, antiviral, antimicrobial, antitumor, and immunomodulatory activities [3]. Oenothein B-rich plant materials, such as willow herb tea (hot extracts from the leaves and flowers of *Epilobium* species of Oenotheraceae), are capable alternatives for use as pharmaceuticals to reduce the risk of diseases closely associated with active oxygen damage [4,5]. These biological effects have been proven using peripheral cells/tissues [2,3], and our in vivo findings showed that oenothein B also functioned in the brain: (1) its per os (p.o.) administration reduced neuroinflammation in the brain during systemic inflammation induced by lipopolysaccharide (LPS; an inflammatory agent) and (2) neuroinflammation-induced abnormal behavior was suppressed in these mice [6].

We also demonstrated that (1) catechol derivatives, such as 4-methylcatechol (4-MC), stimulated the phosphorylation of extracellular signal-regulated kinase (ERK)1/2 along with the neurotrophin receptor Trk B of cultured neurons [7]; (2) the intraperitoneal (i.p.) administration of 4-MC induced an increase in the expression of brain-derived neurotrophic factor (BDNF), a representative neurotrophic factor, in the rat brain [8]. ERK1/2 are important signaling molecules belonging to the mitogen-activated protein kinase (MAPK) family and their signaling pathway has been implicated in diverse cellular events. ERK1 (44-kDa) and ERK2 (42-kDa) exhibit 85% sequence identity and are coordinately activated (i.e., phosphorylated) by various stimuli in many types of cells. In the central nervous system (CNS), ERK2, but not ERK1, has been suggested to function in neurogenesis and cognitive functions [9]. Activated ERK2 leads to the activation of cAMP response element-binding protein (CREB) [10], a transcription factor that is a positive regulator of memory formation and long-term potentiation (LTP) [11]. CREB is also known to function as an important regulator of the expression of BDNF [12] and glial cell line-derived neurotrophic factor [13]. These neurotrophic factors exert neuroprotective effects against neurodegenerative diseases [14].

Based on these findings, we speculated that oenothein B, a large molecular polyphenol, affects the neuronal signaling pathway in the CNS and exerts neuroprotective effects. To clarify the usability of oenothein B-containing plants as herbal medicine for neurodegenerative diseases, we herein examined the effects of p.o. administration of oenothein B on the activation of ERK2/CREB.

## 2. Results

In our previous study on systemically inflamed brains, mice were p.o. administered oenothein B to achieve 100 or 300 mg/kg/day for 10 days [6]. Therefore, the dose in the present study was set to 100 mg/kg/day as well as a higher dosage of 500 mg/kg/day. The duration of its administration in the present study was set to 3 or 7 days. The brain tissue assessed in the immunoblot analysis was the hippocampus because it plays an important role in the consolidation of information from short- to long-term memory [15]. During the experimental period, none of the mice showed any abnormalities in behavior or appetite at either dose.

**Figure 2:**
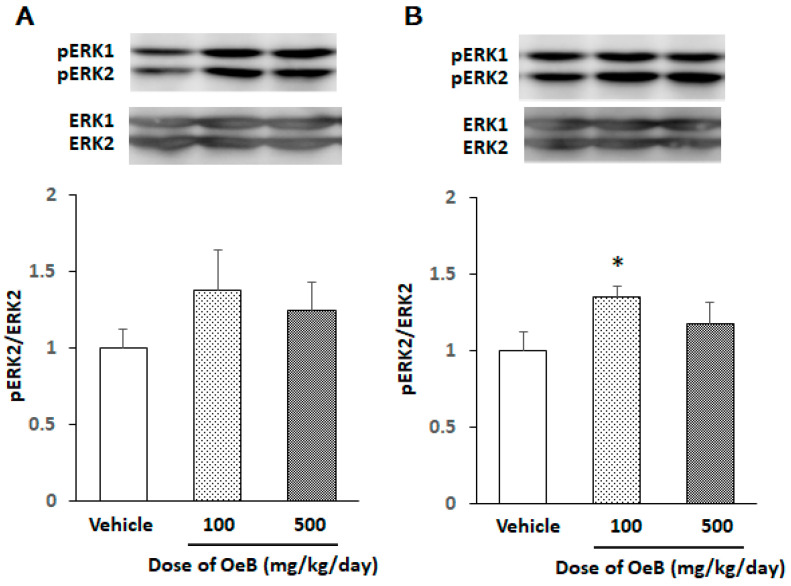
Effects of oenothein B on ERK2 activation in the hippocampal region of mice. Hippocampal tissues prepared on day 3 (**A**) or day 7 (**B**) after the p.o. administration of oenothein B (OeB; 100 or 500 mg/kg/day) or vehicle to mice. The density ratio of phosphorylated components to total components (pERK2/ERK2) in the vehicle-treated group (Vehicle) was expressed as 1.0. Values are means ± SEM (*n* = 5 for each group). Symbols indicate a significant difference as indicated by the brackets: vs. the vehicle-treated group (* *p* < 0.05).

Figure 2 shows the representative bands of a Western blot for total ERK (ERK)1/2 and phosphorylated ERK (pERK)1/2 in hippocampal tissues prepared 3 or 7 days after the administration of oenothein B, indicating that ERK1 and ERK2 were activated by oenothein B almost in parallel. As ERK2 (but not ERK1) isoform has been reported to play a part in the crucial roles in the CNS [9,10], we only analyzed the ratio of pERK2/ERK2 (the vehicle-treated group was expressed as one arbitrary unit). On day 3, the pERK2/ERK2 ratio was slightly but not significantly higher in the oenothein B (OeB)-treated groups than in the vehicle-treated group (Vehicle) (Figure 2A). No significant difference might be caused by a lot of individual variation in the OeB-treated groups. On day 7, the pERK2/ERK2 ratio was significantly (* *p* < 0.05) higher in the OeB (100 mg/kg/day)-treated group but not in the OeB (500 mg/kg/day)-treated group than in the vehicle group (Figure 2B). Oenothein B at the concentration of 500 mg/kg/day might exceed the most suitable concentration.

**Figure 3:**
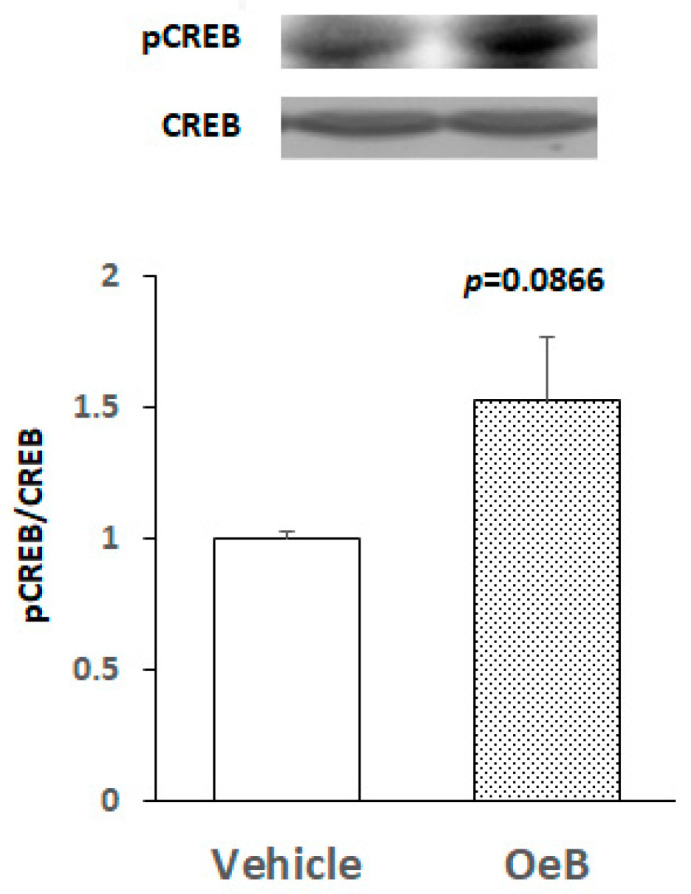
Effects of oenothein B on CREB activation in the hippocampal region of mice. Hippocampal tissues prepared on day 7 after the p.o. administration of oenothein B (OeB; 100 mg/kg/day) or vehicle to mice. The density ratio of phosphorylated components to total components (pCREB/CREB) in the vehicle-treated group (Vehicle) was expressed as 1.0. Values are means ± SEM (*n* = 5 for each group). The Student’s t-test shows that the difference (*p*) between the 2 groups is 0.0866.

To investigate whether the *p.o*. administration of oenothein B induces the activation of CREB in the hippocampus, we examined the tissues of the OeB (100 mg/kg/day)-treated group on day 7 based on the conditions of ERK2 activation. Figure 3 shows the representative bands of a Western blot for total CREB (CREB), phosphorylated CREB (pCREB), and pCREB/CREB ratios, indicating that the pCREB/CREB ratio on day 7 was slightly higher in the OeB (100 mg/kg/day)-treated group (OeB) than in the vehicle group, but no significant difference was observed, contrary to our expectation (*p* = 0.0866).

## 3. Discussion

The present results demonstrated that the p.o. administration of oenothein B activated ERK2 in the healthy mouse brain (Figure 2). The blood–brain barrier (BBB) functions properly in healthy mice. Therefore, hydrophilic oenothein B may not have been able to pass through the BBB under the present experimental conditions. Previous studies showed that ellagitannins are generally transformed in the gut to ellagic acid (2,3,7,8-tetrahydroxy-benzopyrano [5,4,3-cde] benzopyran-5-10-dione), which is then converted to metabolites, such as urolithins (i.e., urolithin A, 3,8-dihydroxyurolithin and urolithin B, 3-hydroxyurolithin), by gut bacteria [16,17,18]. We speculated that any metabolites penetrate and exert their effects in the brain. An important issue that needs to be addressed is identifying which intestinal metabolites of oenothein B affect brain function.

Recent studies showed that ellagic acid has neuroprotective functions in the brain [19,20,21,22]. For example, the subchronic p.o. administration of ellagic acid (100 mg/kg) prevented cognitive and hippocampal LTP deficits and brain inflammation in rats with traumatic brain injury [19]. The chronic i.p. administration of ellagic acid (30 or 100 mg/kg) significantly reversed amnesia induced by scopolamine and antagonized that induced by diazepam [20]. Regarding urolithins, urolithin A has been shown to possess neuroprotective functions [23,24]. For example, urolithin A protected against ischemic neuronal injury in the mouse brain by reinforcing autophagy [23]. Many findings support the widely accepted theory that the effective compound(s) in ellagitannin- and ellagic acid-rich foods are urolithins [25]. However, the chemical structures of the metabolites of oenothein B have not yet been elucidated in detail. We are planning to identify the urinary and plasma metabolites of oenothein B and investigate their abilities to activate ERK2 in future in vivo and in vitro studies.

Another important issue was clarifying the mechanisms underlying the activation of ERK by oenothein B. Since ellagitannins are generally water-soluble, many researchers added oenothein B to the culture medium of various cells in order to investigate their effects [26,27]. In our preliminary experiment, we treated cultured rat cortical neurons with oenothein B and observed the activation of ERK1/2 10 min after the exposure of cells to oenothein B (data not shown). Since oenothein B, a large hydrophilic molecule, cannot pass through cell membranes, its prompt activation of ERK1/2 in vitro suggested that any part of oenothein B binds to corresponding receptors on neurons, which then activate ERK1/2 in the cytosol. We intend to investigate which receptors trigger the activation of phosphorylation cascades by oenothein B in a future in vitro study.

In the present study, we also showed that the p.o. administration of oenothein B activated CREB in the healthy mouse brain (Figure 3). CREB is activated through the phosphorylation of the serine 133 residue by various kinases, such as protein kinase A, Akt/protein kinase B, Ca^2+^/calmodulin-dependent protein kinase, and glycogen synthase kinase-3 [28]. To establish whether the oenothein B-induced phosphorylation of CREB is due to phosphorylated ERK2, further studies using cultured neurons are needed to clarify the effects of U0126 (a specific inhibitor of MAPK/ERK kinase 1; MEK1).

CREB is a positive regulator of memory formation and LTP [11]. We intend to investigate whether the p.o. administration of oenothein B prevents LTP deficits in a temporary amnesia-model mouse. CREB is also the key transcription factor for the expression of some neurotrophic factors [12,13]. These findings suggest that oenothein B exerts neuroprotective effects through the inducible activity of some neurotrophic factors in the brain. Another issue that warrants further study is the inducible ability of oenothein B on the expression of neurotrophic factors in the brain, such as BDNF and GDNF, which will be examined using cultured neurons.

In the last 20 years, vegetable polyphenols, including tannins, have attracted interest in human health care, particularly in terms of the prevention of lifestyle diseases induced by oxygen damage, due to their diverse biological activities that exert strong antioxidant and immunomodulatory effects [29]. The speculation that oenothein B exerts neuroprotective effects through not only its anti-inflammatory activity but also the inducible activity of some neurotrophic factors in the brain may expand pharmacological applications of oenothein B-rich herbs.

## 4. Materials and Methods

### 4.1. Preparation of Oenothein B and Reagents

Oenothein B was isolated from the leaves of *Eucalyptus globulus* (Myrtaceae), as previously described [27,30].

### 4.2. Oenothein B Treatment

Male ddY mice (7 weeks old) were obtained from Japan SLC (Shizuoka, Japan). Oenothein B was dissolved in distilled water and p.o. administered to mice to achieve 100 or 500 mg/kg/day once a day for 3 days (from days 1 to 3) or 7 days (from days 1 to 7). The control group was treated with a vehicle (distilled water). During the experimental period, mice were deprived of food until the administration (10:00) of oenothein B or vehicle (0.3 mL solution) and were then allowed free access to tap water and food until 20:00. Their brains were removed 5 h after the last administration. 

### 4.3. Immunoblot Analysis

Protein extracts of cells and tissues were prepared with a RIPA buffer (20 mM Tris–HCl (pH 7.5), 150 mM NaCl, 0.1% SDS, 1% sodium deoxycholate, 1% NP-40, 2 mM EDTA, and a protease inhibitor cocktail (Roche Diagnostics GmbH, Mannheim, Germany)) as previously described [31]. In immunoblot analysis, protein extracts (10 μg protein) were electrophoresed on SDS-polyacrylamide gels. Proteins were electroblotted onto an Immuno-Blot^TM^ PVDF Membrane (BIO-RAD, Hercules, CA, USA) and reacted with rabbit antibodies against MAPK 1/2 (Erk1/2-CT) (Upstate, Lake Placid, NY, USA), phospho-p44/42 MAPK (Erk1/2) (Thr-202/Tyr-204), CREB, or phospho-CREB (Ser-133) (Cell Signaling, Woburn, MA, USA). The secondary antibody was horseradish peroxidase-linked anti-rabbit IgG (Cell Signaling). Blots were developed using the chemiluminescence method with Plus Western Blotting Detection Reagents (Amersham, Piscataway, NJ, USA).

### 4.4. Statistical Analysis

All results were expressed as means ± SEM. Significant differences in experiments with 2 groups were analyzed using the Student’s t-test. Experiments involving 3 groups were subjected to Dunnett’s multiple comparison test (Prism 6; GraphPad Software, La Jolla, CA, USA). *p* < 0.05 was considered to indicate a significant difference.

## Data Availability

The data presented in this study are available within the article.
